# Challenges in Internet Addiction Disorder: Is a Diagnosis Feasible or Not?

**DOI:** 10.3389/fpsyg.2016.00842

**Published:** 2016-06-06

**Authors:** Alessandro Musetti, Roberto Cattivelli, Marco Giacobbi, Pablo Zuglian, Martina Ceccarini, Francesca Capelli, Giada Pietrabissa, Gianluca Castelnuovo

**Affiliations:** ^1^Department of Literature, Arts, History and Society, University of ParmaParma, Italy; ^2^Psychology Research Laboratory, Ospedale San Giuseppe, IRCCS Istituto Auxologico ItalianoVerbania, Italy; ^3^Department of Psychology, Catholic University of MilanMilan, Italy; ^4^Private PracticeCremona, Italy; ^5^Argo Clinical CenterMilan, Italy; ^6^Department of Psychology, University of BergamoBergamo, Italy; ^7^Private PracticeParma, Italy

**Keywords:** internet addiction disorder, addiction, internet-related psychopathology, diagnosis

## Abstract

An important international discussion began because of some pioneer studies carried out by Young (a) on the internet addiction disorder (IAD). In the fifth and most recent version of the Diagnostic, and Statistical Manual of Mental Disorders (DSM) there is no mention of this disorder and among researchers there are basically two opposite positions. Those who are in favor of a specific diagnosis and those who are claiming the importance of specific criteria characterizing this behavior and the precise role it has in the patient’s life. The aim of the present paper is to answer the question whether it is possible or not to formulate diagnoses of internet-related disorders. We revised literature on the history of diagnostic criteria, on neurocognitive evidence, on the topic debate and on IAD instrumental measures. We found that the disorder was not univocally defined and that the construct was somehow too broad and generic to be explicative for a diagnosis. Indeed, the models are borrowed from other addiction pathologies and they are often formulated before the development of internet as intended in current society. In conclusion, we think we need a more innovative, integrated and comprehensive model for an IAD diagnosis.

## Introduction

One of the biggest problem conceptualizing the psychopathological internet use is that nowadays an important range of activities are carried out online. In fact technological development has radically affected our everyday behavior (Starcevic, 2013). Social relationships have dramatically changed since the time when Young (1996a) begun to assess the internet addiction disorder (IAD). Young (1996a) compared drugs and internet addictions and equated the IAD to the impulse control disorder (Young, 1996a, 1998; Young and Rogers, 1998). For the authors the main point was that both pathologies (IAD and the family of the impulse control disorders) share a common factor: the inability to control the use of something, whether it is internet or a drug. Most importantly, the inability itself interferes with the normal functioning of the individual (Davis, 2001; Young and Rogers, 1998). In seminal studies for this field, Young (1996a) has identified five different kinds of IAD:

1. Cyber sexual addiction (addicted to cyber porn or adult chat rooms);
2. Cyber relationship addiction (cyber affairs or using online relationships to replace real-life friends and family);
3. Net compulsions (obsessive online gambling or shopping);
4. Information overload (compulsive database searches);
5. Computer addiction (obsessive game playing);

Currently many researches are investigating the validity of these categories and the opportunity to include the taxonomy in current diagnostic manuals, in particular the DSM V (Block, 2008; Byun et al., 2009; Aboujaoude, 2010; O’Brien, 2010; Tao et al., 2010; Petry et al., 2014). After heated debated, internet-related pathologies has not been officially included in the DSM V. However, it has been mentioned as an entity that needs further investigation and research (American Psychiatric Association, 2013). Researchers willing to list the pathology in the DSM (e.g., Block, 2008) are claiming that the IAD shows enough criteria to be included in the substance dependency family.

The aim of the present paper is to revise literature in order to understand whether it is possible or not to formulate diagnoses of internet-related disorders. Our hypothesis is that the IAD categorization by Young, formulated in the 1990s, is not that specific to take into consideration how internet use has evolved in the last decades. Nowdays it is difficult to determine what is pathological with internet use. Differently from 20 years ago, we use internet for a very broad spectrum of daily activities such as to work, to have fun, to sell and buy, to socialize and so on. Thus, a model that compares internet with drug abuse is not sufficient to explain the current heterogeneity of this behavior.

## History of IAD Diagnosis Criteria

Young (1996a) purposed that IADs would share the common seven criteria among other addiction disorders as pathological gambling (Griffiths, 2000), eating disorders (Lacey, 1993; Lesieur and Blume, 1993), sexual addictions (Goodman, 1993), generic technological addictions (Griffiths, 1995), and video game addiction (Griffiths, 1998). The seven criteria would be: (i) withdrawal, (ii) tolerance, (iii) preoccupation with the substance, (iv) heavier or more frequent use of the substance than intended, (v) centralized activities to procure more of the substance, (vi) loss of interest in other social, occupational, and recreational activities, and (vii) disregard for the physical or psychological consequences caused by the use of the substance. Considering these seven-criteria model for IAD diagnosis, authors developed a eight-items questionnaire (IADQ – Addiction Diagnostic Questionnaire; Young, 1998) to provide a screening instrument for IAD identification. In a single case study, Young (1996b) indicates also two risk criteria increasing the probability to develop IAD: (i) the type of application used on internet; (ii) the “high” sense of excitement when using internet. On one hand, Young made a tentative to compare IAD with other addictions, but on the other hand, she recognized that specific criteria are necessary for the quantification of the problem.

On a similar note, other researchers (Beard, 2005; Block, 2008) compared IAD to other behavioral addictions, but they noted four common criteria among addictions: (i) excessive use, associated with loss of sense of time or neglect basic drivers; (ii) withdrawal and feelings of anger or depression when the computer is not available; (iii) tolerance (more hours of use to satisfy the need); and (iv) negative social repercussion. The latter matched Young’s criterium for withdrawal, social consequences, and quantity of time spent on internet. Similarly, Shapira et al. (2000) defined the use of internet as problematic when it implies maladaptive preoccupation, irresistible or protracted periods longer than planned. Finally, Orzack (1999) wrote about “the pathological use of the computer” when the computer is utilized as a means to satisfy, induce excitement, and reduce tension or induce relief, matching the excitement needing guessed by Young as a risk criterium.

We think that these pioneer attempts to find IAD criteria are very precious and explicative but, given the velocity of the development of internet use in the actual society, they cannot take into consideration the real current use of internet instrument and they risk to be over-inclusive if used to conceptualize DSM pathological criteria today. A more recent contribution by Tao et al. (2010) suggested a more complex frame for the diagnosis. Authors explained that, today, all the following criteria should be considered for a complete diagnostic inclusion in the IAD category: (i) preoccupation with the internet; (ii) withdrawal symptoms; (iii) tolerance; (iv) unsuccessful attempts to control internet use; (v) continued excessive internet use despite knowledge of negative psychosocial problems; (vi) loss of interests, previous hobbies, entertainment as a result of, and with the exception of, internet use; (vii) use of the internet to escape or relieve a dysphoric mood; and (viii) deception of family members, therapists, or others.

To summarize, criteria considered as fundamental as a base for a diagnosis of IAD are, today, a matter of debate. We observe a great disagreement on the criteria definitions of IAD, particularly, on what makes internet use pathological or not, i.e. quantitative criteria such as the time spent on internet or the degree of preoccupation about it or qualitative criteria such as dysphoric mood or maladaptive preoccupation about it (Chou et al., 2005; Dell’Osso et al., 2006; Widyanto and Griffiths, 2006; Shaw and Black, 2008; Weinstein and Lejoyeux, 2010; Hinic, 2011; Tonioni et al., 2012; Kuss et al., 2014; Spada, 2014).

## Assessment Instruments

Despite the lack of a common theoretical and diagnostic model, assessment methods have been developed for IAD since the first studies in the 1990s. The most popular tests are indeed the Addiction Diagnostic Questionnaire (IADQ) and the internet addiction test (IAT; Young, 1998). The latter and other principal instruments are listed in **Table 1**.

**Table 1:** Assessment instruments for internet addiction.

Study	Instrument	Diagnostic criteria	Structure
Young, 1998	Addiction Diagnostic Questionnaire	Pathological gambling criteria	8 items; measure scored dichotomously
Young, 1998	Internet Addiction Test	Dependency criteria	20 items 1- to 5- Likert-scale
Chen et al., 2003	Chen Internet Addiction Scale	Internet addiction	26 items 4-point Likert-scale
Caplan, 2002	Generalized Problematic Internet Use Scale (GPIUS)	Based on Davis’ (2001) cognitive-behavioral model of problematic Internet use	29 items self-report questionnaire rated on 5-point Likert-scale
Demetrovics et al., 2008	Problematic Internet Use Questionnaire (PIUQ)	Focused on obsession, neglect, and control disorder	30 items scored on a 5-point Likert-scale


The only two instruments that properly investigate internet addiction are the IAT (Young, 1998) and the Chen Internet Addiction Scale (Chen et al., 2003). The former has been built on Young’s model focusing on the dependency as a central factor and the aim of the questionnaire is to provide a score of pathology. The latter was born as a screening instrument for IAD in adolescents and follows DSM-V diagnostic criteria. The other instruments investigate more general problematic internet-related behaviors and do not allow a diagnosis of the disorder.

The most extended limitation among these tools was their self-report nature, tough, in some cases, they also measured the online activity (Coman and Sillitti, 2009). Moreover, several items are becoming obsolete. Time spent over the internet is in general increasing among users and establishing “the right” amount of time to spend in front of a screen could be problematic. Measures in this field are likewise becoming more complicated especially taking under consideration the use of the internet on mobile devices. In conclusion, we can assert that current methods used to assess and measure the IAD are not satisfying and they are lacking an unanimous consensus (Chou et al., 2005; Dell’Osso et al., 2006; Demetrovics et al., 2008; Meerkerk et al., 2009; Wallace and Masiak, 2011; Pezoa-Jares et al., 2012; Laconi et al., 2014).

## Neuroscientific Evidence for IAD Diagnosis

From a neurocognitive point of view, the literature on IAD is not clearer. There are very few studies on subjects with IAD diagnosis. The main process that seems impaired in IAD patients is the decision-making process (Sun et al., 2009). Dong et al. (2013) demonstrated that IAD subjects need longer time than control subjects to take decision during an internet activity.

However, all the studies focusing on neural correlates generalize their results from a specific internet behavior to the whole IAD category. Particularly the internet gaming disorder (IGD) has been studied. An increase in the gray matter density in the left ventral striatum was evident in experimental subjects (teenagers with IGD) relative to healthy subjects belonging to the control group (Hong et al., 2013). By contrast, other authors found a reduction in the density of gray matter in several regions such as the dorsolateral prefrontal cortex, the orbitofrontal cortex, the motor area, cerebellum, and the left rostral anterior cingulate cortex (Yuan et al., 2013). Moreover, a reduction of the gray matter in the anterior cingulate cortex, the insula and in both the posterior and the orbitofrontal cortex was evident (Zhou et al., 2011; Hong et al., 2013; Yuan et al., 2013). A study on gaming disorder with the resting-state fMRI found a reduction of connectivity among the right inferior temporal gyrus, the bilateral parietal cortex, the posterior cingulate cortex, as well as among the cingulate gyrus and the rear right precuneus, part of the thalamus, caudate, ventral striatum and supplementary motor areas (Ding et al., 2013). Taken together these results suggest that, in the IGD, specific areas for cognitive control, motor control and reward processes are implied.

Despite the interesting implications for IAD comprehension, we cannot consider IGD brain activations as explicative for IAD. The IGD construct is too specific. It implies only one internet activity and it is far from the current pervasive use of internet in our everyday life. On the other hand, what emerges from the IGD neuroscientific literature is an involvement which is a too spread of control process areas that are not distinct from areas involved in control processes for other disorders or other addictions.

We think that the lack of an articulated model of IAD complicates neuroscientific studies on the phenomenon. A better definition of IAD and its specific components is required, in terms of kind of stimulus or activity (i.e. game, social), instruments (i.e., PC and tablet), settings (work and fun), and potential criteria that could distinguish between pathological or not. Besides the time spent on internet or the degree of preoccupation considered for the diagnostic criteria two neurocognitive studies suggest that the level of excitement provoked by the internet stimulus could be a good discriminant criterium. Two studies (Laier et al., 2013a,b) underlined the strong appeal internet stimulus have on subjects affected with IAD, compared to the low attractiveness that non-internet-related stimulus have on the same subjects.

To summarize, neuroscientific literature on IAD is untimely and it is affected by the lack of a good model for the diagnosis that should give more precise methodological indications for the choice of settings, paradigms, and measures.

## Comorbility

There is clear evidence of high rates of psychiatric comorbidity with the IAD among both adolescents and adults (for a review Spada, 2014). IAD is associated with major psychiatric conditions and dysfunctional personality traits (Jiang and Leung, 2012): neuroticism (Tsai et al., 2009; Yan et al., 2013); psychoticism (Yan et al., 2013); sensation, and novelty-seeking (Park S. M. et al., 2012); impulsivity (Cao et al., 2007; De Berardis et al., 2009; Lee et al., 2012); high level of aggressiveness (Ko et al., 2009b).

Moreover, further associations have been found as impairment of daily life functioning (Lam et al., 2009; Young, 2009; Fu et al., 2010; Tao et al., 2010) and reduction of psychological wellbeing (Ni et al., 2009). Whang et al. (2003) demonstrated correlations between social exclusion and IAD. Neverthless, a causal effect that links social isolation with an internet dependency and vice-versa has not been found (Chou et al., 2005).

In absence of a defined classification of the IAD, it seems to be impossible to isolate clinical conditions associated with the dependency itself. Currently, only one study describes moderate empirical evidence of the IAD as a stand-alone condition (Fu et al., 2010). The paper considers the prevalence of IAD in Hong Kong adolescences and authors made an effort to differentiate it from other correlates such as anxiety and depression.

In conclusion, researchers are still not keen on considering the IAD a well-defined nosological classification. The literature of comorbility with IAD is abundant and we described here the more salient ones. We report a list of papers, grouped by disorders, in **Table 2**.

**Table 2:** Comorbility for internet addiction disorder.

Clinical conditions	Studies
Attention deficit hyperactivity disorder	Yoo et al., 2004; Ha et al., 2006; Ko et al., 2008, 2009a; Bernardi and Pallanti, 2009; Yen J. Y. et al., 2009; Griffiths, 2012; Gundogar et al., 2012; Chou et al., 2015
Depression	Young and Rogers, 1998; Shapira et al., 2000; Kim et al., 2006; Yen et al., 2007; Ko et al., 2008; Lee et al., 2008; Bernardi and Pallanti, 2009; Yen C. F. et al., 2009; Morrison and Gore, 2010; Xiuqin et al., 2010; Cheung and Wong, 2011; Tsitsika et al., 2011; Gundogar et al., 2012; Park J. W. et al., 2012
Hypomania and bipolar disorders	Shapira et al., 2000; Bernardi and Pallanti, 2009
Anxiety	Black et al., 1999; Bernardi and Pallanti, 2009
Obsessive–compulsive disorder	Pies, 2009; Carli et al., 2013
Substance abuse	Bai et al., 2001; Lam et al., 2009; Korkeila et al., 2010; Ko et al., 2012
Suicide risk	Mathy and Cooper, 2003; Kim et al., 2006
Dissociative symptoms	Bernardi and Pallanti, 2009; De Berardis et al., 2009; Canan et al., 2012
Insomnia	Xiuqin et al., 2010
Alexithymia	De Berardis et al., 2009; Yates et al., 2012; Dalbudak et al., 2013
Low self-esteem	Armstrong et al., 2000; De Berardis et al., 2009; Fioravanti et al., 2012


## Toward or Against an IAD Diagnosis?

After revising literature on diagnosis and instruments about IAD, we note that there is difficulty with a definition of the problem itself. Internet is no longer a simple means of communication, but it represents a necessary way of living in the professional, academic, and sentimental life. The introduction of mobile devices (smartphones, tablets, etc.) has also radically changed the way people connect (Hinic, 2011). Thus, internet use is no longer limited to a stable place in front of a PC, but pretty much everywhere (De Vries, 2013). Then, we can actually say that connection time, one of the more prominent criteria of all conceptualization attempts, is no more a variable able to distinguish between pathological and not pathological internet use (Del Miglio et al., 2001; Tonioni et al., 2012; Wallace, 2014).

There is a debate between eastern scientists that are more oriented to accept the diagnosis due to the higher number of cases and western scientists that are quite critical on the validity of the IAD construct (Mitchell, 2000; Shaw and Black, 2008; Manjikian, 2012). Currently, in the DSM-V only the IGD can be found. Critical issues are targeted to methodological aspects but also to conceptual features (Widyanto and Griffiths, 2006; Yellowlees and Marks, 2007). Bell (2007) stated that “Internet dependency” is itself a wrong term. It cannot be defined as an addictive behavior since the internet is just a mean of communication and not a behavior as such. Internet is nowadays part of the environment, ubiquitous and able to modify cultural beliefs (Starcevic, 2013). An alternative is to investigate which activities are associated with the IAD (Kuss et al., 2014).

Cantelmi et al. (2000), expressing doubts on the mainstream definition of the internet dependency, proposed a different one named internet-related psychopathology (IRP). In their understanding, these clinical conditions are not an internet dependency but a group of specific psychopathological situations consumed online. These can be typically: pathological gambling, cyber-sex, game dependency, information overload addiction (Cantelmi et al., 2000). An assessment tool that has been proposed to identify IRP pathology is the IRP-AS (Cantelmi and Talli, 2009), a software able to measure the online activity of the subject. The program measures several qualitative and quantitative aspects: connection time, online actions and frequency of exposure to specific content (pornography, gambling, and gaming).

Other authors distinguished among three different “internet-related activities” (Starcevic, 2013), suggested another term for the disorder (“internet-mediated psychopathology” – Tonioni, 2013). and criticized the current IAD concept and proposed the compensation model (Kardefelt-Winther, 2014). A further attempt toward the development of the IAD diagnosis was the work by Karaiskos et al. (2010) who included a wider set of symptoms and called IAD as Internet Spectrum Dependency.

Considering the debate exposed thus far, we think that we are far from IAD precise diagnostic criteria. Our position is that we need a theoretical model proper of IAD functioning in current époque before formulating diagnostic criteria. Thus, basic research should go in the direction of observing the phenomenon on different levels such as neurophysiological, neurocognitive, psychological, and sociological level, which should be integrated to build a comprehensive theory on internet use behavior.

## Alternative to IAD Diagnosis

The ubiquity of internet has a massive impact on social behaviors. These changes have been investigated by several disciplines and scientific points of view. Psychology and psychiatry themselves have probed this relatively new field. If the improvement of digital technology has had a large impact on redefining the meaning of human relations, it is nearly impossible to ignore the implications on the definition of a healthy versus pathological condition (Silver and Massanari, 2006; Barak, 2008). We must emphasize that psychopathologies are not absolute entities and they vary over time and, more important, are influenced by the historical and cultural context. The IAD seems to be a category heavily linked to past experiences that are not up to date with current internet usage. De Kerckhove (1995, p.5) states “telephone, radio, television, computers and other media combine to create environments that, together, establish intermediate realms of information processing”. New technologies are reorganizing the mental and the relational architecture of contemporary human beings (De Kerckhove, 1995). For this reason, in a world in which it is likely to be progressively online, research should focus on investigating behaviors that may be viewed as pathological considering the cultural changes that affect our modern society (Kirmayer et al., 2013). At present, it seems quite a simplification to consider the Internet as an object or a situation, or a tool that can be used or abused as other substances. Internet is an omnipresent aspect of human interaction. Therefore, it should be advisable to update our concept of the IAD and talk about a web-mediated psychopathology instead (Tonioni, 2013).

It is still unclear whether web-users can become addicted to the internet or on the internet (Griffiths, 2000) and whether the dependency is structured on a more specific activity (i.e., pathological online gambling). However, the position can be reversed by assuming that the internet is becoming itself a primary need.

Consistently with this perspective, Jurgenson (2012) proposed to overcome the dualism between real versus digital introducing the concept of augmented reality. Currently, internet use is not a “different reality” (immersive and synthetic) to daily reality, but a part of everyday life itself. The distinction between real life (offline) and virtual reality (online) as well as the basis for the categorization of IAD are too simplistic. The diversity of online activities is neglected by the diagnostic criteria of IAD. Therefore, in line with other authors (Starcevic, 2013) we believe that this category should be abandoned as the internet has become a wide umbrella term. “In some ways, saying someone is addicted to the internet is akin to arguing that somebody with a drinking problem is addicted to a liquor store” (Van Rooij and Prause, 2014, p. 204). Today, more complete and convincing categorizations are advancing. Specifically, the concept of IRP (Cantelmi et al., 2000; Tonioni, 2013) depicts a more appropriate vision of the constellation of disorders related to the excessive internet use. This new concept considers a wide constellation of clinical conditions linked to the web. Each internet addiction, such as cyber-sex, cyber-relationships, gaming, gambling, or information overload would be a specific pathology and it shares with others only the use of online canals. This position would allow studying each pathology, in terms of diagnosis, instruments and neural correlates, in an independent way in order to take into account the complexity that internet phenomenon is reaching today.

## Discussion and Conclusion

We reviewed literature on IAD to understand whether we are near or far from a diagnosis of internet-related disorders was reviewed this paper. After considering current principal models, history of diagnostic criteria, neuroscientific and quantitative measures literature, our position is that we are far from a comprehensive model on internet use that does not limit to explain the disorder as a simple addiction but that conceptualizes the behavior in the light of current cultural implication of internet use in our everyday life. Our opinion is that a comprehensive model to explain internet-related behaviors should be more articulated and multilevel relative to the models available now. First, we cannot consider internet addiction exclusively as other kind of addictions such as to drugs. Even if they have some points in common, they cannot share the same diagnostic criteria since the use of internet is today necessary and pervasive and it could be employed in a normal way. This is not the case of drugs. Moreover, we need a better investigation of criteria that we should consider to determine the pathological versus non-pathological use of internet. The time spent on internet, in current time, is not sufficient as discriminant criterion. The influence of psychological criteria such as the preoccupation about the absence of the activity or the level of excitement during internet stimulation should be better investigated. A model considering internet behaviors as an articulated constellation of independent disorders, joined only by the internet use, should be appropriated for the heterogeneity of the phenomenon. In other words, we should go in a direction where internet-related behaviors should assume an independent identity among each other and the use of internet itself should became a normal tool we must use to survive but which can be utilized in several pathological ways.

## Author Contributions

AM: Substantial contributions to the conception of the work, deep analysis of the literature, development and final approval of the paper. RC and MC: Contribute to the development and revision of the work, with deep literature analysis and agreement for approval of the paper. MG, FC, and GP: Contribute to the development and revision of the work, with literature analysis and agreement for final approval of the paper. PZ: Contribute to the development and revision of the work, substantial proposal of neuropsychological support for the model, with literature analysis and agreement for final approval of the paper. FC: Contribute to the development and revision of the work, with literature analysis and agreement for final approval of the paper. GC: Contribute to development and deep revision of the work, with literature analysis and agreement for final approval of the paper.

## Conflict of Interest Statement

The authors declare that the research was conducted in the absence of any commercial or financial relationships that could be construed as a potential conflict of interest.
